# Investigation and Assessment for an effective approach to the reclamation of Polycyclic Aromatic Hydrocarbon (PAHs) contaminated site: SIN Bagnoli, Italy

**DOI:** 10.1038/s41598-019-48005-7

**Published:** 2019-08-08

**Authors:** Carmine Guarino, Daniela Zuzolo, Mario Marziano, Barbara Conte, Giuseppe Baiamonte, Lorenzo Morra, Daniele Benotti, Davide Gresia, Edoardo Robortella Stacul, Domenico Cicchella, Rosaria Sciarrillo

**Affiliations:** 1 Department of Science and Technology, University of Sannio, via Port’Arsa 11, 82100 Benevento, Italy; 2 Invitalia Via Calabria, 46 Roma, Italy

**Keywords:** Abiotic, Environmental impact

## Abstract

Native plant species were screened for their remediation potential for the removal of Polycyclic Aromatic Hydrocarbons (PAHs) contaminated soil of Bagnoli brownfield site (Southern Italy). Soils at this site contain all of the PAHs congeners at concentration levels well above the contamination threshold limits established by Italian environmental legislation for residential/recreational land use, which represent the remediation target. The concentration of 13 High Molecular Weight Polycyclic Aromatic Hydrocarbons in soil rhizosphere, plants roots and plants leaves was assessed in order to evaluate native plants suitability for a gentle remediation of the study area. Analysis of soil microorganisms are provides important knowledge about bioremediation approach. *Alphaproteobacteria*, *Betaproteobacteria*, *Gammaproteobacteria* are the main phyla of bacteria observed in polluted soil. Functional metagenomics showed changes in dioxygenases, laccase, protocatechuate, and benzoate-degrading enzyme genes. Indolacetic acid production, siderophores release, exopolysaccharides production and ammonia production are the key for the selection of the rhizosphere bacterial population. Our data demonstrated that the natural plant-bacteria partnership is the best strategy for the remediation of a PAHs-contaminated soil.

## Introduction

“Intense industrial activities in the 20^th^ century has been particularly deleterious to the environment, resulting in a large number and variety of contaminated sites”. Furthermore, once these industrial areas have been abandoned, the contamination with substances that are dangerous for the environment and for human health remains. Heavy Metals (HMs), PolyChloroBiphenyls (PCB) and Polycyclic Aromatic Hydrocarbons (PAHs) can pollute former industrial area. All these compounds can be formed both by anthropogenic activities and by natural emissions. Anthropogenic origins include petrogenic and pyrolytic (which represents the main process responsible of PAHs contamination) origin. Pyrolytic origins comprise partial combustion of organic matter (such as fossil fuels and biomass) mainly occurring during industrial and other human activities. While the petrogenic PAHs are constitute by petroleum products, such as accidental oil spills, discharge from routine tanker operations, municipal and urban runoff^1,2^.

For all these reasons, PAHs are among the most commonly studied groups of organic pollutants frequently found in abandoned industrial areas. These compounds are environmentally persistent with various structures (two or more fused benzene rings) and varied toxicity. They represent an important health risk since they can enter the food chain (resulting in bioaccumulation and biomagnification). In soils, PAHs have a low mobility and high durability and their amount depends of different factor as temperature, pH, and soil organic matter content^3,4^ and ageing of the history of contamination in soils^5^. Nowadays brownfields recovery sites represents a challenging issue throughout the world, which has gained increasing attention in recent years in order to prevent contaminants migration and to allow redevelopment^6,7^. At the same time, brownfields are attractive for site planning (since they are often situated in areas adjacent to the central parts of the cities)^8^.

**Figure 1:**
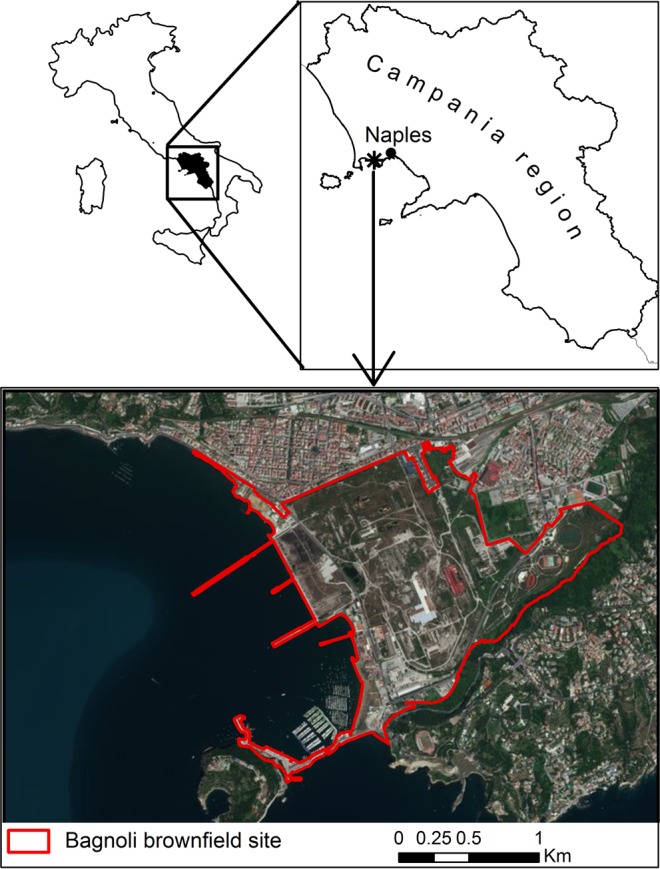
Location map of the study area (Bagnoli brownfield site, Southern Italy). From Google Maps (https://www.google.com/maps/place/80124+Bagnoli+NA/@40.8171802,14.1516033,5444m/data=!3m1!1e3!4m5!3m4!1s0x133b0e97616d81eb:0xb53d31e14d5424cd!8m2!3d40.8171821!4d14.1691129).

Bagnoli, abandoned industrial area, represent one of the main brownfield site in Italy. Its steel plant (overlooking the Gulf of Naples) was for a century, until its demise in the mid-1990s, Italy’s largest one (Fig. 1). Unluckily, the products and by-products of industrial processes changed the natural equilibrium environment^9^, so its remediation has become a priority. Among the different remediation technologies, an *in-situ* bioremediation approach has been chosen and is ongoing to clean-up soils of Bagnoli brownfield site. This bioremediation strategy uses live plants (woody or herbaceous plant) and microorganisms populations that naturally live in the soil that are adapted to the contamination to recover the polluted areas; is non-destructive and environmentally/eco-friendly and represent an interesting research field, which results has been considerably documented over the last decades^8,10,11^. Different bacteria, naturally present in soil, are able to degrade PAHs as alpha, Beta and Gamma *Proteobacteria*, *Bacteroidetes*, *Actinobacteria*, *Firmicutes*^12^. Among *Protobacteria*, *Betaproteobacteria* are the best to PAH degradation^13,14^. *Burkholderiales* are especially well known to degrade PAHs^15,16^. In addition, *Gammaproteobacteria* are able to degrade PAHs^17^.

The main objectives of this study are: (*i*) discrimination of contamination levels and origins of PAHs in the analysed soils; (*ii*) native PAHs tolerant plants identification; (*iii*) identification of natural bacterial communities in PAHs contaminated soils; (*iv*) identification of their Plant Growth Promoting (PGP) attributes; (*v*) identification of main enzymes produced by native microbiota of a soil polluted with PAHs; (*vi*) selection of the better system for phytomanagement of the study area.

## Results and Discussion

### Distribution of PAHs in rhizosphere soils

**Figure 2:**
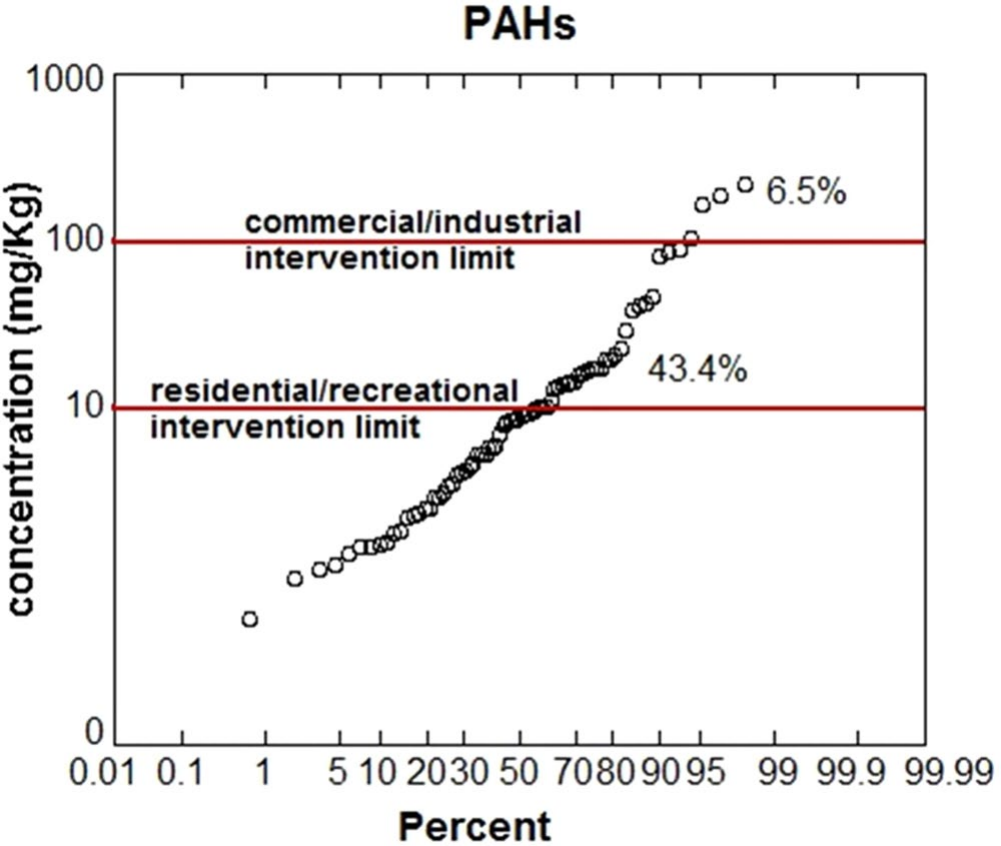
Probability distribution and local density of soil ∑PAHs data produced by site characterization (set up by Italian Government). The X-axis is scaled in probability (between 0 and 100%) and shows the percentage of the Y variable whose value is less than the data point. The Y-axis displays the range of the data variables.

**Figure 3:**
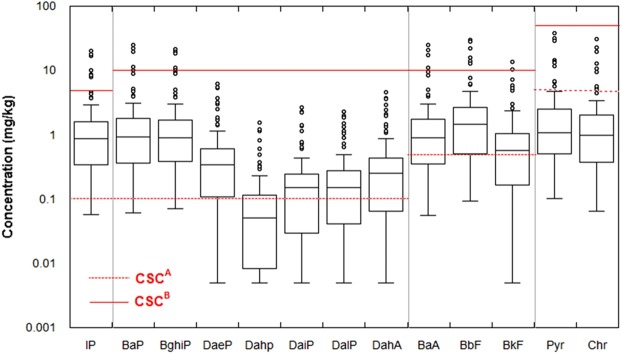
PAHs congeners’ concentration levels of soil PAHs data produced by site characterization (set up by Italian Government). Dashed and continued lines correspond to threshold limit for residential/recreational and commercial/industrial land use (Legislative Decree 152/06), respectively.

The Italian legislation (Legislative Decree 152/06) provide two threshold limits for ∑PAHs: 10 and 100 mg/kg for recreational and commercial land use, respectively. Data produced within this study (set up by Italian Government) show that 43.4% of the analysed soils are characterized by ∑PAHs above the residential/recreational limit (Fig. 2); also, the commercial/industrial land use threshold is exceeded by a percentage of 6.5% of total samples. Based on Italian Government remediation objective (which correspond to residential/recreational land use recovery of the area) site contamination is mainly governed by higher molecular weight PAHs congeners BghiP > BaP > IcdP (Fig. 3). In addition, the commercial/industrial threshold limit is exceeded by several congeners (except for DaeP, DaiP, DalP, DahA, Pyr and Chr) (Fig. 3). Table 1 listed the mean statistical parameters of the measured PAHs concentrations in the rhizosphere soil. Our results showed that the mean concentrations of Pyr (3.37 ± 0.78 mg/kg), BbF (3.32 ± 0.66 mg/kg), Chr (2.68 ± 0.59 mg/kg), BaP (2.43 ± 0.53 mg/kg) and BaA (2.34 ± 0.51 mg/kg) were significantly higher in rhizosphere soils. The concentration levels of analysed rhizosphere soils reveal a strong PAHs pollution. They were among the highest ones compared to the literature reported values for other contaminated soils of other areas in the world (Table S1- Supplementary Material), which suggest a strong anthropogenic input from industrial activities on the study area.

**Table 1:** Measured concentration of PAHs (mg/kg) in the rhizosphere soil from Bagnoli brownfield site.

Compound	Rizhosphere soils			
	Min	Max	Mean	SE
*BaA*	0.055	24.7	2.34	0.51
*BaP*	0.061	24.8	2.43	0.53
*BbF*	0.094	30.0	3.32	0.66
*BkF*	0.005	13.7	1.35	0.28
*BghiP*	0.07	21.7	2.32	0.48
*Chr*	0.064	31.0	2.68	0.59
*DahA*	0.005	4.6	0.54	0.10
*IP*	0.057	20.2	2.14	0.44
*Pyr*	0.101	38.0	3.37	0.78
*DaeP*	0.005	6.3	0.75	0.14
*Dahp*	0.005	1.54	0.14	0.03
*DaiP*	0.005	2.7	0.29	0.06
*DalP*	0.005	2.32	0.30	0.05
∑ *PAHs*	0.5746	221.5	21.9	4.70

Instrumental Detection Limit (IDL) is 0.01 mg/kg. For statistical computation, data below the instrumental detection limit (IDL) were assigned a value corresponding to 50% of the detection limit.

#### PAHs sources

**Figure 4:**
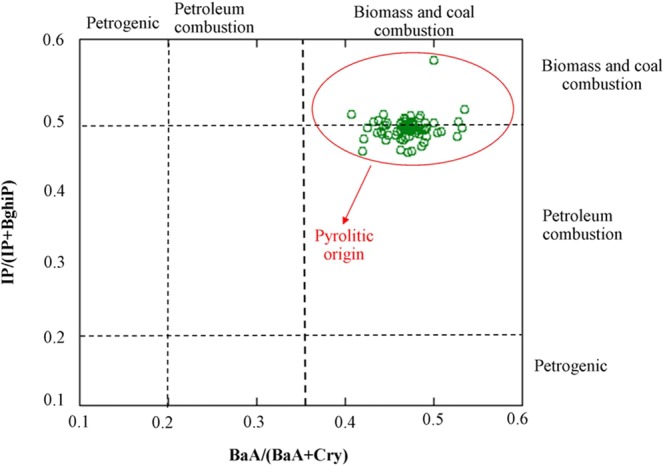
Cross-plots for the isomeric ratios of *IcdP/(IcdP* + *BghiP)* versus *BaA(BaA* + *Chr)* for soil PAHs source characterization.

The combined use of the diagnostic ratios allowed us to PAHs origin of the investigated area. BaA/(BaA + Chr) ratios ranged from 0.40 to 0.53, presenting ratios characteristic of pyrogenic source. This is also supported by looking at IcdP/(IcdP + BghiP) ratios, ranging from 0.44 to 0.57 for which origins of PAHs are more likely to be related to coal/biomass and petroleum combustion. Indeed, the analysed soils show an unmistakable pyrogenic origin (Fig. 4) related to past industrial processes including dust, ash, carbon coke residues, heavy oils, hydrocarbons, and combustion residues. Fingerprint of coal combustion as reported by Vivo and Lima^9^ is clearly highlighted by adopted diagnostic ratios.

### PAHs in the plants system

**Figure 5:**
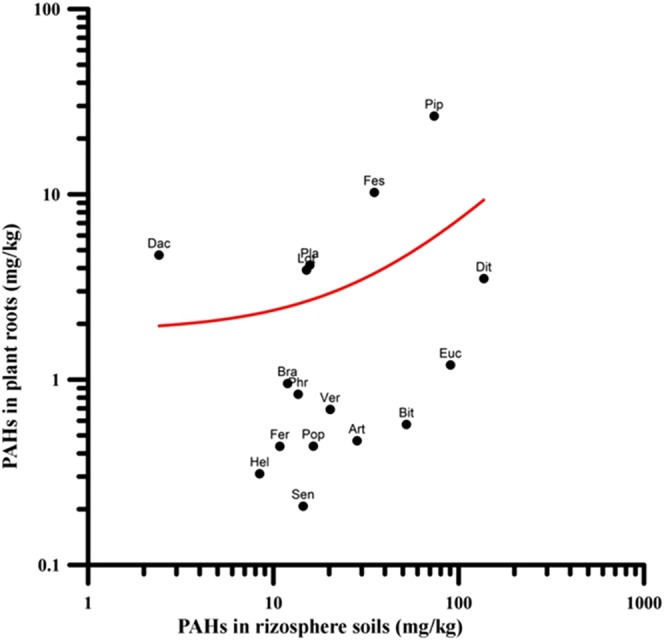
Scatterplot showing the relationship between ∑PAHs concentration in the rhizosphere soils and in plant roots for selected native species – data represent the mean value of 3 replicates.

**Figure 6:**
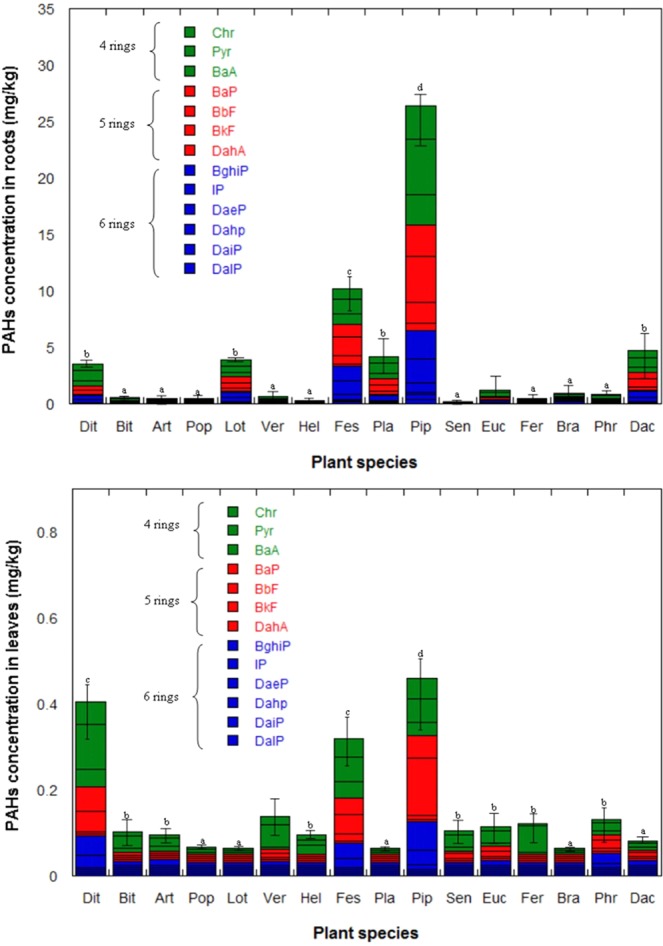
PAHs concentrations (mg/kg) in roots and leaves. Columns with the same letter are not significantly different at p < 0.05, according to ANOVA-protected Tukey’s post hoc test. Error bars indicate Standard Error (SE).

The families more present in the Bagnoli brownfield site are Poaceae (20 taxa, 14.4%), Fabaceae (15 taxa, 10.8%), Asteraceae (14 taxa, 10.1%) and Apiaceae (6 taxa, 4.3%). These plants are adapted and able to survive and reproduce under contaminated soil condition^18^. PAHs concentrations in the plant system (root and leaves) were determined for 16 native plant species. Pearson correlation analysis suggested a positive correlation (with a Pearson coefficient of 0.63 and 0.64 at p < 0.001, respectively) between PAHs content in plants roots and leaves and the related rhizosphere soil. The scatterplot of Fig. 5 highlights that roots accumulation is promoted by both soil contamination levels and plant species. Figure 6 highlight PAHs accumulation in roots and leaves respectively by analysed plant species. The different profiles of PAHs in plant tissues indicated that the 4-ring and 5-ring PAHs mostly contributed to total concentration of PAHs in both roots and leaves. Higher molecular weight PAHs are generally incapable of translocation to the aboveground plant parts because of their low water solubility, and they are strongly bound to the roots^19^. An organic element enters a plant roots for the presence of the lipid in a plant that, even at small amounts, is usually the principal source for highly water-insoluble contaminants^20^. Lin *et al*.^21^ demonstrated that the storage of PAHs in maize plant (*Zea maize L*.) was directly congruent to the lipid level in tissues that allows the free diffusion within cells.

In a recent studies^22,23^ shown that seeds of *Zea mays* are ecotoxicity indicator on soils contaminated with petro derivates.

As the PAHs, level in leaves is roughly not significative; our interest must focus on root system. Among the analysed plant species, *Pip* and *Fes* show the highest ΣPAHs root concentration, with mean content of 26.4 and 10.2 mg/kg, respectively. However, *Dac* demonstrates to be the best root bioaccumulator. In addition, *Lot* and *Dit* show promising ΣPAHs root phytostabilization rates (Fig. 6). *Pip* has suitable attributes like as fast growth and high root cover; in addition, it is able to stimulate soil microbial communities^24,25^.

The considerable PAHs content in roots was not only imputable to the contamination by adherent soil particles. However, as suggested by Fismes *et al*.^26^ it is possible that part of the PAHs measured in plants roots can be due to their adsorption on the roots epidermis. “*In fact*, *root peels are mainly made of suberin*, *a polyester with phenolic and aromatic functions presenting a lipophilic pole*^27^ *able to strongly adsorb PAHs*^28,29^”.

### Soil bacteria community

**Figure 7:**
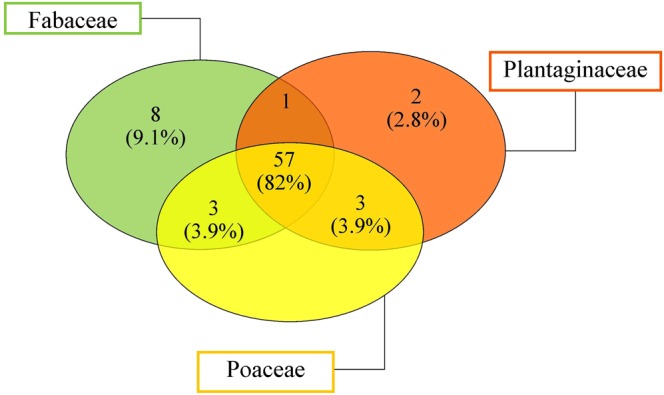
Venn-diagram showing the intersection of isolated bacteria from rhizosphere soils of the three main plant families found in Bagnoli brownfield site (Fabaceae, Plantaginaceae and Poaceae).

A natural bioremediation process is defined by the rating of bacterial functional and structural diversity in soil directly from the contaminated site. Rhizosphere soil collected from three different plants (*Pip*, *Lot* and *Pla*) that are present in Bagnoli brownfield were characterized by abundant biodiversity for approximately 93–97% of the total biodiversity in each sample. The Venn diagram for the bacterial populations of the three-rhizosphere soils of three different plants is shown on Fig. 7. In the soils collected directly from three plants (*Pip*, *Lot* and *Pla*), 82% of the bacterial communities were found to be in common. Therefore, the microbial community composition were highly heterogeneous. The analysis of soils showed that within *Proteobacteria*, *Alphaproteobacteria* and *Gammaproteobacteria* were the taxa with higher proportions, both containing known species of PAH degraders and *Actinobacteria* populations as well. *Firmicutes* and *Deltaproteobacteria* were relatively less of all other groups (Table 2). All three rhizosphere soils investigated showed *Gammaproteobacteria* as the most abundant class with *Pseudomonadaceae* family. In literature is known the use of *Pseudomonas* genera employed in bioremediation study of soil polluted by PAHs^30^ reported that the degradation of complex mixtures could be done using a pool of bacteria with different functions and capacity to utilize as source of carbon and energy the hydrocarbons^31–33^. Because the endophyte communities is mainly present in phylum *Proteobacteria*^34^, we hypothesize that our identified bacteria, belonging for 82% to phylum of *Proteobacteria*, could act as endophytes. In Table 2 there are the main genera of bacteria that behave like endophytes, *Pseudomonas*, *Bacillus*, *Burkholderia*, *Rhizobium* and *Microbacterium*^34–38^. A good method to reduce crude oil is a biodegradation process via cometabolism. Gałazka *et al*.^30^ and Doong and Lei^39^ reported that in case of cometabolism hydrocarbons could not be a font of carbon and energy but has function as co-substrates, so their degradation is due by the presence of diverse microorganisms (e.g. parathion is cometabolized by *Pseudomonas stutzeri* to 4-nitrophenol and diethylphosphate, and phenol is then used as a source of carbon and energy by *P*. *aeruginosa*); the researcher conclude that cometabolism is one of the most important mechanism in the transformation of PAHs in soil. *Pseudomonas aeruginosa* make PAH-oxidative enzymes and release rhamnolipids^40^. *Bacillus cereus* degrade both LMW and HMW PAHs^41,42^. Besides, Cavalcanti *et al*.^43^ emphasizing the adjunct of consortia composed by *Pseudomonas aeruginosa* and *Burkholderia cepacia* strains in the removal of phenanthrene and pyrene from a soil contaminated by a lubricating oil mixture containing PAH. In polluted soil the presence of *Alphaproteobacteria*, family as *Bradyrhizobiaceae* (slow-growing rhizobia), *Rhizobiaceae*, *Nitrobacter* and *Sphingobium*, known in literature to do nodules on the roots of leguminous plants and fixing nitrogen, is important. Komaniecka *et al*.^44^ called “bacteroids” the endosymbiotic rhizobia, in which nitrogen fixation takes place. All bacterial species isolated in this study are known to be a good PAHs degradation^45^. Different paper reported the import role of bacterial consortium. Vaidya *et al*.^46^ reported the use of *Pseudomonas*, *Burkholderia* and *Rhodococcus* (PBR) was able to degrade 99% of pyrene under microcosm conditions. Other consortiums like *Pseudomonas sp*. and *Bacillus sp*. isolated from oil sludge that are able to reduce total petroleum hydrocarbons from 63 to 84% in six weeks^47^; and the consortium composed of *Pseudomonas aeruginosa* and *Rhodococcus sp*. isolated from soil polluted with oily sludge demonstrated 90% degradation of hydrocarbons in 6 weeks^47^. Plant roots release exudates as sugars, organic acids, fatty acids, secondary metabolites, nucleotides and inorganic mixture that perform an important key in establishing and determining the rhizosphere microbial population^48^. These exudates determine and regulate directly or indirectly the activity of biodegradative microorganisms. In this study, the capability of isolates strain to make indole-3-acetic acid, siderophores, exopolysaccharides and ammonia was evaluated (Table 2). Thirty-nine of the isolates were able to produce Indolacetic acid (IAA). All three-rhizosphere soil had *Paenibacillus polymyxa* as the best IAA producing. Both in Poaceae and in Plantaginaceae rhizosphere soil *Rhizobium leguminosaurum* produced highest amount of IAA. *Mycobacterium sp*. MOTT36Y, instead, produced only in Poaceae family highest amount of IAA. Rhizosphere of Plantaginaceae and Fabaceae had *Pseudomonas cichorii* as good IAA producer. Plantaginaceae and Fabaceae showed respectively typical strains with high IAA *Paenibacillus sp*. JDR-Z, *Bacillus cereus* group, *Klesbiella aerogenes* for the first and *Rhizobium etli bv mimosae* for the second one. In this study, the 60% of isolates were found to have ability for siderophores production. *Rhodococcus jostii* produced in all three-rhizosphere soils high amount of siderophores; only in Plantaginaceae, also *Bacillus cereus* group showed siderophores activity. The three-rhizosphere soils showed 50% of isolates capable of producing EPS. The high amount of EPSs production was present in *Burkholderia cenocepacia*, *Burkholderia ambifaria* AMMD, *Bacillus cereus* group, *Sinorhizobium fredii*, *Sinorhizobium meliloti*. Only the 38% of isolates displayed the production of ammonia. The high amount of ammonia was in *Burkholderia pseudomallei* NCTC 13178 and *Bacillus cereus* group.

**Table 2:** Primary screening of the assessment of potential PGP by bacteria isolates recovered from rhizosphere soil of *Piptatherum miliaceum*, *Lothus corniculatus* and *Plantago lanceolata*.

Bacterial isolates	Phylum	Class	Order	Family	Genus	Production IAA	Siderophore release	EPSs production	Production of ammonia
*Bradyrhizobium japonicum USDA6*	Proteobacteria	Alphaproteobacteria	Rhizobiales	Bradyrhizobiaceae	*Bradirhizobium*	++	−	−	++
*Bradyrhizobium japonicum*						+++	+++	+++	+++
*Bradyrhizobium diazoefficiens*						++	++	++	+
*Bradyrhizobium oligotrophicum*						++	++	+	+
*Bradyrhizobium oligotrophicum S58*						+	−	+	−
*Nitrobacter hamburgensis*					*Nitrobacter*	−	—	−	−
*Nitrobacter hamburgensis X14*						−	−	−	−
***Rhizobium leguminosaurum***				Rhizobiaceae	*Rhizobium*	+++	+++	+++	+++
***Rhizobium leguminosarum bv trifolii***						++	++	++	++
***Rhizobium leguminosarum bv trifolii WSM2304***						++	++	++	++
***Rhizobium etli CIAT652***						+	+	+	+
***Rhizobium etli bv mimosae***						−	++	−	−
***Sinorhizobium fredii***					*Ensifer*	+++	+++	+++	+++
***Sinorhizobium meliloti***						−	++	−	−
***Mesorhizobium ciceri***				Phyllobacteriaceae	*Mesorhizobium*	−	−	−	−
*Achromobacter xylosoxidans*		Beta Proteobacteria	Bukholderiales	Alcaligenaceae	*Achromobacter*	++	++	−	−
*Burkholderia cenocepacia*				Burkholderiaceae	*Burkholderia*	+	−	−	−
*Burkholderia ambifaria AMMD*						−	+	+	—
*Burkholderia pseudomallei NCTC 13178*						+	−	−	++
*Burkholderia gladioli*						+	−	−	+
*Burkholderia glumae BGR1*						−	−	−	−
*Paraburkholderia rhiroxinica*					*Paraburkholderia*	−	−	++	++
*Paraburkholderia xenovorans*						+	+	−	−
*Pseudomonas aeruginosa*		Gammaproteobacteria	Pseudomonadales	Pseudomonadaceae	*Pseudomonas*	+	+	−	−
*Pseudomonas aeruginosa PA7*						−	++	−	−
*Pseudomonas stutzeri*						−	+++	−	−
*Pseudomonas mendocina NK-01*						++	++	−	−
*Pseudomonas resinovorans*						++	++	−	−
*Pseudomonas resinovorans NBRC 106553*						+++	−	−	−
*Pseudomonas fluorescens*						+++	+++	+++	+++
*Pseudomonas fluorescens Pf0-1*						−	−	−	−
*Pseudomonas fluorescens SBW25*						−	++	++	−
*Pseudomonas fluorescens F113*						−	++	++	−
*Pseudomonas poae*						−	+	+	−
*Pseudomonas putida*						−	+	++	−
*Pseudomonas putida H8234*						−	+	++	−
*Pseudomonas putida NBRC 14164*						−	−	−	−
*Pseudomonas putida GB-1*						−	−	−	−
*Pseudomonas putida HB3267*						+++	−	−	−
*Pseudomonas fulva 12-X*						+	−	−	−
*Pseudomonas stutzeri DSM 10701*						+	−	−	−
*Pseudomonas stutzeri DSM 4166*						+	+	+	
*Pseudomonas stutzeri A1501*						+	+	−	+
*Pseudomonas syringaepv*.*tomato*						++	++	++	−
*Pseudomonas brassicacearum*						++	++	++	−
*Pseudomonas pertucinogena group*						++	++	++	−
*Pseudomonas cichorii*						+++	+	++	−
*Enterobacter aerogenes*			Enterobacteriales	Enterobacteriaceae	*Klesbiella*	++	−	+++	+
*Geobacter bemidjiensis*		Deltaproteobacteria	Desulfuromonadales	Geobacteriaceae	*Geobacter*	++	−	+++	+
*Rhodococcus opacus B4*	Actinobacteria	Actinobacteria	Actinomycetales	Nocardiaceae	*Rhodococcus*	++	−	−	+
*Rhodococcus jostii*						+	++	+	+
*Rhodococcus pyridinivorans sb3094*						++	++	++	++
*Mycobacterium abscessus subsp bolletii*				Mycobacteriaceae	*Micobacterium*	++	+	++	+
*Paenibacillus mucilaginosus K02*	Firmicutes	Bacilli	Bacillales	Paenibacillaceae	*Paenibacillus*	++	++	++	++
*Paenibacillus mucilaginosus 3016*						+	+	+	+
*Paenibacillus sp JDR-2*						−	−	−	−
*Paenibacillus polymyxa*						+	++	++	+
*Bacillus cereus group*				Bacillaceae	*Bacillus*	+++	++	++	++
*Bacillus thuringiensis serovar finitimus*						++	−	−	−
*Deinococcus proteolyticus*	Deinococcus-Thermus	Deinococci	Deinococcales	Deinococcaceae	*Deinococcus*	−	−	−	−

In bold the bacteria isolates typical of *Lothus corniculatus*, in underline those typical of *Plantago lanceolata*. The isolates were categorized into three groups according to the produced amount: +low concentrations (<1 µg/ml), ++moderate concentrations (1–2.99 µg/ml) and +++high concentrations (>3 µg/ml).

### Functional analyses of PAH degrading genes

**Figure 8:**
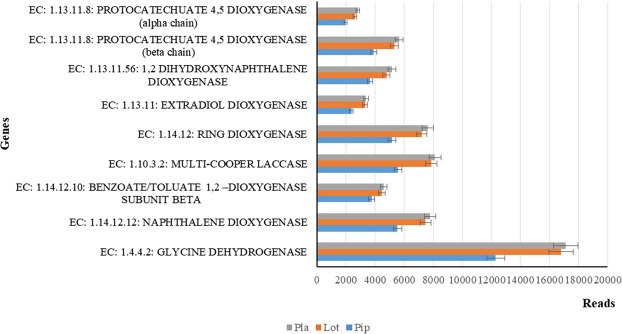
Genes for degrading PAHs from soil metagenomes of *Piptatherum miliaceum* [Pip], *Lothus corniculatus* [Lot], *Plantago lanceolata* [Pla].

The reads and fragments (in million) produced in three different rhizosphere soils are: 0.45 M of fragments and 0.91 M of reads in *Piptatherum miliaceum*, 0.57 M of fragments and 1.15 M of reads in *Lothus corniculatus*, 0.57 M of fragments and 1.14 M of reads in *Plantago lanceolata*. In contrast with the heterogeneity of the taxonomy of bacteria structure (82%) in three different rhizosphere soils, there are important alterations in the abundance of the genes encoding enzymes for PAHs degradation (Fig. 8). Our results showed a high gene abundance of several oxidoreductases involved in PAH metabolism, as a test of the PAH degradation potential by native soil microorganisms^11,49–51^. In fact, the dioxygenase family genes (naphthalene 1,2-dioxygenase, extradiol dioxygenase, benzoate 1,2 dioxygenase, protocatechuate 4,5-dioxygenase (alpha and beta chain) and 1,2-dihydroxynaphthalene dioxygenase) are the most abundant in PHAs contaminated soils, particularly in rhizosphere soil of *Plantago lanceolata* as compared to *Piptatherum miliaceum* and *Lothus corniculatus* (Fig. 8). Therefore, from our data, it is possible to hypothesize different ways of degradation of the PAHs, although all could start by an initial oxidation by laccases followed the activity of dioxygenases and decarboxylases, helping the ulterior degradation of PAHs; hypothesizing that natural soil bacterial populations are able to use specific enzymes and common pathways to degrade PAHs.

## Conclusions

Our obtained data showed that PAHs contamination at the Bagnoli brownfield site (Southern Italy) is relevant and its origin is mainly due to the industrial activities and processes that had been working for decades. Almost the 44% of the soil samples in the industrial district were contaminated by PAHs with 4, 5, and 6 ring. Media contents of ∑PAHs in the soil were higher than the maximum permitted concentrations for residential/recreational land use, as it represent the remediation objective stated by Italian Government for Bagnoli site recovery. All PAHs congeners overcomed the recommended limits for corresponding soil categories and BghiP, BaP and IP mainly control pollution. Analysis of diagnostic ratios showed that petroleum and coal combustion were the main sources of PAHs in the investigated site. The studied uptake by native plants indicates that PAHs root accumulation is promoted and movement to the aboveground biomass (translocation) is limited. Interesting PAHs roots accumulation rate were observed for several native species. Based on our results, plant PAHs concentration levels mainly depend on rhizosphere soil PAHs content and type of plant species. Maximum PAHs root accumulation was found in monocotyledons Poaceae such as *Pip* and *Fes*. These type of plants are known to have a fibrous root system with several moderately branching roots growing from the stem. While, a taproot system is common in dicotyledons. Monocots fibrous root system consisting of a mass of similarly sized roots that maximizes absorption. Boyle and Shann^52^ showed that also microbial activity was higher in monocot rhizosphere soils than dicot ones soils and demonstrated that monocot rhizosphere soil degraded many organic contaminants faster than dicot soils. *Piptatherum miliaceum* (L.) Coss on has suitable attributes such as rapid growth and high root cover and stimulates soil microbial communities^24^. Besides, our data indicated that Rhizoremediation process involve both plants and their associated rhizosphere microbes. Various microbes include as *Pseudomonas aeruginosa*, *Pseudomonas fluoresens*, *Mycobacterium* spp., *Rhodococcus* spp., *Paenibaciluus* spp. are involved in PAHs degradation^48,53^. Our bacteria that for about 85% belong to the phylum of *Proteobacteria* in general have a marked propensity to the endophytic habitat. This is very important both for determining the performance of the plant and for the degrading activity of their rhizosphere^34^. Functional metagenomics showed that the abundance of genes degrading PAHs is present in three rhizosphere soils, confirming the metabolic potential of PAHs-degrading microorganisms present into contaminated soil, and assuming a possible ways of degradation PAHs in contaminated soils^11,12^. The root-exudates help the activity of the microbes, which respond to the exudate, buffet rapidly during the bioremediation of soils. This is in concordance with previous works in which it was markedly noted a strong rhizosphere effect on degradation of PAHs through the stimulation of indole-3-acetic acid, siderophores, exopolysaccharides and ammonia. Our data show that PAHs pollution selects a specific rhizosphere microflora that degrades and helps the plant to adapt better and therefore to high performance. Therefore, these results can lead to innovative biotechnological applications in bioremediation processes in the field. In conclusion, our results demonstrated that the microbe-assisted phytoremediation was found to be the most advantageous approach obtaining high rates of degradation of PAHs in relation to other strategies, demonstrating that *Piptatherum miliaceum*, *Lothus corniculatus* and *Plantago lanceolata* association could be an environmentally sound management approach for the treatment of aged PAH-polluted soils.

## Materials and Methods

### Site description and sampling

The Bagnoli brownfield area (2 km^2^; situated in correspondence of Phlegrean Fields between 40°49′30″91 and 40°47′30″North, and 14°9′30″ and 14°12′0″ East) falls into the western part of the city of Naples (Campania region), Southern Italy. This industrial district was one of the most important integrated steelworks in Italy during the last century until its closure in the nineties due to economic and environmental reasons. Because of industrial activities, large amount and types of contaminants were generated and dispersed throughout the whole area. The sampling campaign was conducted during 2017 with the identification of 76 areas characterized by high concentrations of PAHs in which the dominant plant species were identified. The most represented species are shown in Fig. S1 (Supplementary Materials). Roots and leaves of each species were gathered and stored in sterile polypropylene tubes. Rhizosphere soil samples of the above plants were also gathered and stored in polypropylene tubes and kept at 4 °C for further analysis. All analysis were performed in triplicate.

### Soil and plant (roots and leaves) PAHs analyses

The analysis of PAHs comprised of 13PAHs and followed the procedure US-EPA method 8270D. The target analytics (Table 3) were extracted by an accelerated solvent extractor, purified using a silica gel column, and detected by a gas chromatograph (7890 A, Agilent, USA) coupled with a mass spectrometer (5977B, Agilent, USA). Deuterated fluorene, Deuterated fenanthrene, Deuterated chrysene and Deuterated perylene were added to the samples prior to extraction, and were considered as internal standards for quantification of the 13 PAHs as described by the USEPA^54^.

**Table 3:** List of PAHs analysed and their abbreviations, also used in the text and figures.

Polycyclic Aromatic Hydrocarbons PAHs	Abbreviation	Molecular weight (g/mol)	N° of rings
Chrysene	Chr	228	4
Pyrene	Pyr	202	4
Benzo[a]anthracene	BaA	228	5
Benzo[a]pyrene	BaP	252	5
Benzo[b]fluoranthene	BbF	252	5
Benzo[k]fluorantene	BkF	252	5
Dibenzo[a,h]anthracene	DahA	278	5
Benzo[g,h,i]perylene	BghiP	276	6
Indeno [1,2,3-c,d] pyrene	IP	276	6
Dibenzo[a,e]pyrene	DaeP	302	6
Dibenzo[a,h]pyrene	Dahp	302	6
Dibenzo[a,i]pyrene	DaiP	302	6
Dibenzo[a,l]pyrene	DalP	302	6

The number of rings and molecular weights are also reported.

At every 20 samples were enclosed the determination of a certified reference material (Beechwood (PCP and PAH) BCR® certified Reference Material- Sigma-Aldrich, Italy) such as quality control. Blanks and matrices plus standard addition (a mixture of 13 EPA PAHs and 4 Deuterated PAHs) were quantified sporadically to determine the accuracy of the testing. Procedural blanks were quantified sporadically to evaluate the cross-contamination. In the blank controls the PAHs elements were under the limits of detection.

### Soil PAHs fingerprint

Since PAHs emission profile for a given origin can be conditioned by the processes producing the PAHs^55^, diagnostic ratios are a largely used to apportion the origin of PAHs. According to previous reports^56–58^ the ratios of BaA/(BaA + Chr) and IcdP/(IcdP + BghiP) have been adopted. Because very low proportions of BaA or IP are rarely observed in combustion samples, a BaA/(BaA + Chr) or IcdP/(IcdP + BghiP) ratio less than about 0.20 likely indicates natural petroleum-related source. A BaA/(BaA + Chr) ratio value from 0.20 to 0.35 indicate petroleum combustion and >0.35 imply biomass and coal combustion. Literature values suggest biomass and coal combustion for a IcdP/(IcdP + BghiP) ratio above 0.50. While, combustion products of gasoline, kerosene, diesel and crude oil all have ratios below 0.50, with vehicle emissions falling between 0.24 and 0.40.

### Selection of the native bacteria

The rhizosphere soils were selected of three species of three different families that showed high uptake of PAHs: *Piptatherum miliaceum* (L.) Coss. (Poaceae)[*Pip*], *Lothus corniculatus* L. (Fabaceae) [*Lot*], *Plantago lanceolata* L. (Plantaginaceae) [*Pla*]. For the selection, 1 g of homogenized rhizosphere soil was incubated in duplicate in 50 ml of a mineral medium at pH 6.8 with the addition of 5% of a mixture of PAHs (EPA 610 Polynuclear Aromatic Hydrocarbons Mixture, Sigma- Aldrich, Milan Italy)^10^.

Brief as described in Guarino *et al*.^10^, cultures were incubated in 250 ml of a mineral medium in conical flasks at 28 °C in an orbital shaker (200 rpm) for five days. Then, to isolate the greatest number of strains, 100 ml of serial tenfold dilutions of bacterial cultures were propagated on two different solid media: LB and R2A (Sigma-Aldrich, Milan Italy) for to not overlook slower growing colonies. First colonies were visualized after 4 days of incubation at 28 °C and after other five days, a total count of colonies was performed^10^. For each rhizosphere soil have been obtained and preserved fifty-one colonies per medium and per samples were randomly selected and maintained as pure cultures^10^.

### Molecular identification of native isolated strains of bacteria

Two grams of rhizosphere soil of three species of three different families were used for the isolation of genomic DNA using the PowerSoil DNA Isolation Kit (MoBio Laboratories Inc., USA) from following the manufacturer’s instructions. Extracted DNA preparations were quantified and quality checked using a Nanodrop 1000 Spectrophotometer. Universal eubacterial primers (F27a: AGAGTTTGATCCTGGCTCAG; R1492a: GGTTACCTTGTTACGACTT) were used as template for16S rRNA gene amplification. Polymerase chain reaction (PCR) were performed with GoTaq® Polymerase (Promega) according to the supplier’s instructions. PCR-amplified DNA was sequenced with a BigDye® Terminator v3.1 Cycle Sequencing Kit (Applied Biosystems Inc. USA) using an automated DNA sequencer (ABI model 3500 Genetic Analyzer). Nucleotide sequences were edited and assembled with Lasergene version 11.2.1 (DNASTAR®) and subjected to homology comparison (BLAST analysis) at the National Center for Biotechnology Information (NCBI) server (www.ncbi.nlm.nih.gov/blast/Blast.cgi)^10^.

### *In vitro* tests for plant growth promoting (PGP) traits

The bacteria strains were analysed for Plant Growth Promoting activities (PGP). Indolacetic acid (IAA) production, siderophores release, exopolysaccharides (EPSs) production and ammonia production were determined as described by Guarino *et al*.^10^.

### Library preparation and sequencing PHAs degrading genes

‘Nugen Ovation Ultralow System V2′ kit (Nugen, San Carlos, CA) was used for library preparation. The samples were quantified and quality tested using the Qubit 2.0 Fluorometer (Invitrogen, Carlsbad, CA) and Agilent 2100 Bioanalyzer (Agilent Technologies, Santa Clara, CA). Libraries were processed and sequenced on MiSeq (Illumina, San Diego, CA), pair-end with 300 cycles per read. Base calling and demultiplexing were performed on instrument. Analysis were focused on the genes involved Polycyclic Aromatic Hydrocarbons (PAHs) degradation as KEGG elements reported in a recent work^11^. Gene sequences were downloaded from Uniprot, and the short reads obtained from the sequencing experiments were blasted against the above-mentioned genes using BLASTX v2.2.29. All reads resulting in a hit with an e-value lower than 0.1 were retained for analysis.

### Statistical data analyses

Univariate statistical analyses were performed to show the single-element distribution. The data below the instrumental detection limit (IDL) were assigned a value corresponding to 50% of the detection limit^59^. The Pearson correlation coefficients between PAHs concentrations in plants and rhizosphere soils were calculated. The statistical significance of the results was verified at the significance level of alpha < 0.05.

## Supplementary information

Table S1 and Figure S1
