# Supplementary material for: Investigation and Assessment for an effective approach to the reclamation of Polycyclic Aromatic Hydrocarbon (PAHs) contaminated site: SIN Bagnoli, Italy
Source: Sci Rep. 2019 Aug 8;9:11522. doi: 10.1038/s41598-019-48005-7 (PMC6687822; doi:10.1038/s41598-019-48005-7)
Supplement: Supplementary file 1 — Table S1 and Figure S1 [file 41598_2019_48005_MOESM1_ESM.pdf]

Investigation and Assessment for an effective approach to the reclamation of Polycyclic Aromatic Hydrocarbon (PAHs) contaminated site: SIN Bagnoli, Italy

Carmine Guarino, Daniela Zuzolo, Mario Marziano, Barbara Conte, Giuseppe Baiamonte, Lorenzo Morra, Daniele Benotti, Davide Gresia, Edoardo Robortella Stacul, Domenico Cicchella, Rosaria Sciarrillo

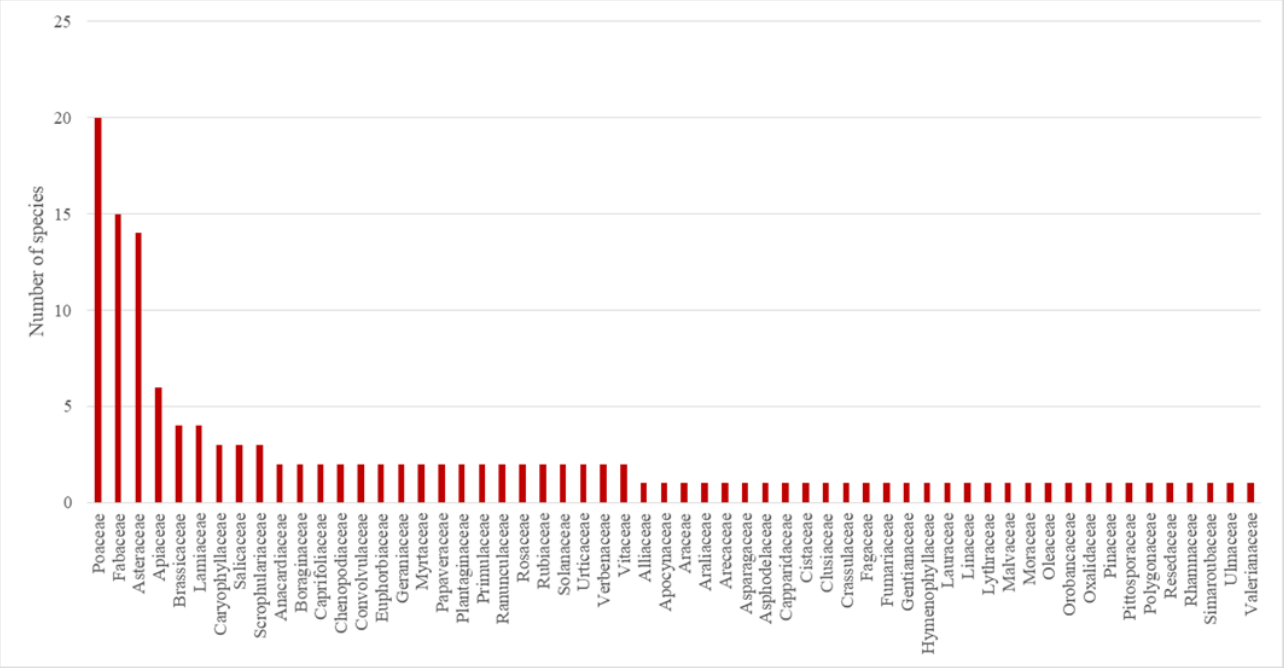

Biological spectrum of recorded plant species in Bagnoli site. Number of species per family in Bagnoli site.

Investigation and Assessment for an effective approach to the reclamation of Polycyclic Aromatic Hydrocarbon (PAHs) contaminated site: SIN Bagnoli, Italy

Carmine Guarino, Daniela Zuzolo, Mario Marziano, Barbara Conte, Giuseppe Baiamonte, Lorenzo Morra, Daniele Benotti, Davide Gresia, Edoardo Robortella Stacul, Domenico Cicchella, Rosaria Sciarrillo

	Min	Mean	Max	Location	References
∑PAHs	-	-	300	50 meters from an oil refinery (Zelzate, Belgium)	Bakker et al. (2000)
	<0.02	13.5	769.6	Stassfurt (Germany)	Krauss and Wilcke (2003)
	12	-	50	Mosel and Saar river bank soils (Germany)	Pies et al. (2008)
	0.082	-	45.02	Delhi (India)	Bhupander et al. (2012)
	0.29	2.99	8.49	Brownfield site in Shenyang (China)	Sun et al. (2013)
	0.21	1.19	2.61	Smelting site (Chenzhou, China)	Sun et al. (2014)
	0.88	4.29	32.47	Coke power site (Chenzhou, China)	Sun et al. (2014)
	4	18	67	London (UK)	Vane et al. (2014)
	0.007	-	0.75	Urban areas (Southern Italy)	Thiombane et al. (2018)
	0.0019	-	11.35	Rural areas (Southern Italy)	Thiombane et al. (2018)
	1.8	34.1	187.1	Rizosphere soils from Bagnoli brownfield site	This study

Comparison of PAHs concentrations (mg/kg dry weight) throughout areas in the world.
